# Multi-Objective Spatial Prioritisation for Protected-Area Network Expansion in Xinjiang, China

**DOI:** 10.3390/biology15201863

**Published:** 2026-10-16

**Authors:** Lei Han, Zhi Wang, Yayan Lu, Xinsheng Zhu, Luyao Wang, Shuaishuai Han, Hui Shi

**Affiliations:** 1 College of Animal Science and Technology, Henan University of Science and Technology, Luoyang 471023, China; hanlei@haust.edu.cn; 2 School of Business, Henan University of Science and Technology, Luoyang 471023, China; 9906174@haust.edu.cn (L.W.); sshan@haust.edu.cn (S.H.); 3 College of Tourism, Henan Normal University, Xinxiang 453007, China; luyayan16@mails.ucas.ac.cn; 4 Research Institute of Forest Resource Information Techniques, Chinese Academy of Forestry, Beijing 100091, China; daben1989@126.com; 5 College of Tourism, Xinjiang University of Finance and Economics, No. 449 Beijing Road, Urumqi 830012, China

**Keywords:** conservation hotspots, conservation conflict, wilderness, restoration and rewilding, landscape connectivity

## Abstract

Establishing protected areas (PAs) in the right locations is essential for the Kunming–Montreal Global Biodiversity Framework’s 30% target, yet existing PA networks are often misaligned with conservation needs. We used systematic conservation planning to identify priority areas in Xinjiang, China, under four objectives, biodiversity, landscape connectivity, ecosystem services, and their integration, by minimising a composite human-pressure cost while meeting quantitative representation targets, and connectivity was linked to priority setting by ranking patches by their contribution to overall landscape connectivity. The four solutions each covered 30% of Xinjiang but overlapped on 19.76% of the region, and no single objective satisfied all targets. The extant PA network was poorly aligned with these priorities; only 47.10–51.25% of its area fell within priority areas and 77.22–79.06% of priority areas lay outside existing PAs, leaving range-restricted species such as the Ili pika (*Ochotona iliensis*) (0.10% protected) largely uncovered. Most additional priority areas were expansion zones adjoining existing PAs, with a few large patches contributing disproportionately to connectivity. We therefore propose a tiered pathway: expand existing reserves, secure high-connectivity bridges, refine small patches through ground surveys, and complement by restoring small patches in human-modified landscapes with community participation and fiscal-transfer support.

## Simple Summary

Xinjiang, in the west of China, hosts many rare species, but its nature reserves do not always protect the places that have high biodiversity. Here, we identified the priority areas for conserving biodiversity, keeping natural habitats connected, and preserving the ecosystem services. We found that focusing on any single goal leaves many species unprotected, so all goals should be considered together. We also found that only about half of the existing reserves overlap the priority areas, while more than three-quarters of these priority areas lie outside current reserves, leaving species such as Ili pika (*Ochotona iliensis*) almost entirely unprotected. We therefore suggest expanding existing reserves first, connecting them with corridors, and then restoring small patches of nature in farmland and settled areas, with the participation of local communities.

## 1. Introduction

Changes in land use, which may lead to habitat loss, fragmentation, and degradation, have been identified as major drivers of biodiversity loss [1,2,3]. Protected areas (PAs), such as national parks and nature reserves, are regarded as a core strategy for mitigating the adverse impacts of the expansion and intensity of human-induced stressors on biodiversity and ecosystem services [4]. PAs contain higher levels of biodiversity, lower pressures of human disturbance and habitat conversion, and provide more robust ecosystem services delivery than nearby unprotected sites [5,6,7]. Although protected and conserved areas covered more than 17% of global land by 2020 [8], this is widely considered insufficient to halt biodiversity loss [9]. The Kunming–Montreal Global Biodiversity Framework (GBF) Target 3 calls for effectively protecting at least 30% of land and sea by 2030, using PAs and other effective area-based conservation measures (OECMs), especially areas of particular importance for biodiversity and ecosystem functions and services (the 30 × 30 goal) [10], with this pledge already endorsed by 124 countries (https://www.hacfornatureandpeople.org/, accessed 15 August 2026). To meet this goal, a large number of areas will be designated as PAs or OECMs over the next decade, raising a fundamental question: where should new PAs be located, according to which objectives, and how should they be configured?

In the practice of the expansion of PAs, a persistent challenge is the spatial mismatch between protected lands and conservation need. Historical and ongoing designation has consistently preferred selecting remote, high elevation and areas without or with minimal human disturbance [11,12]. The lower societal opportunity costs of these wilder areas and their lower suitability for agriculture and development will deliver intact ecosystem processes and buffer species against land-use pressures, as well as afford more opportunities for the future expansion of PAs, whereas human-dominated landscapes present greater challenges. However, species richness and biodiversity hotspots are disproportionately concentrated in human-dominated landscapes: 32.8% of global protected land was under intense human pressure [13], and less than 3% world’s biodiversity hotspots and 2% of threatened vertebrate concentration areas are free of human pressure [14]. As PA expansion has historically followed the path of least resistance toward remote wilderness, it has created persistent gaps in species coverage, particularly for threatened taxa and ecosystems. In fact, the places which offered the greatest biodiversity benefits are precisely those most likely to be ignored. New PAs should accordingly be located in both remote wilderness and human-dominated landscapes, with the management models of the IUCN management categories matched to each: strict wilderness reserves (Category Ib) for near-pristine lands, and protected landscapes or sustainable-use reserves (Categories V and VI), which integrate conservation with continued human activity, for working landscapes [15]. Therefore, a broader spatial perspective is essential for addressing the persistent mismatch between where new PAs are most easily established and where they are most urgently needed.

Addressing this spatial mismatch requires systematic spatial prioritisation. In mapping global or regional priority areas, some studies directly focused on species richness and endemism [16,17,18,19], whereas others have highlighted the aspects that limit further impacts of human disturbance or climate change on biodiversity, proposing that sites with integral ecosystems [20], high ecosystem services [21,22], and ecological connectivity [23], and sites that are large in size [24], should be prioritised. Prioritisation maps built on different objectives frequently partially overlap or conflict, for instance, prioritising on intactness alone (e.g., wilderness or high-integrity forests) can halve extinction risk [25], yet such areas often harbour relatively low endemism and thus misalign with species-centred priorities [12]. Therefore, networks designed for a single objective may undermining the broader crucial aspects of the GBF vision, e.g., biodiversity, ecosystem functions, and the services they provide to people [9,26]. Indicating that the PA expansion works should confirm the intended achievement, and how multiple conservation objectives can be reconciled within a single spatial framework.

Apart from as a measurable conservation objective of individual sites, connectivity could also be seen as a property of the network as a whole, which only appeared once a site was added to the existing conservation network. Indeed, even for a well-chosen site that meets multiple conservation objectives, the value that it delivers depends on where it sits in the wider PA network. Isolated PAs cannot prevent the extinction debts produced by the isolation of different populations and their habitats [27,28]; networks that lack connectivity impede dispersal, gene flow, and climate-driven range shifts, and may turn into future ecological traps from current reserves [29]. Connectivity has accordingly been considered to be an explicit requirement of global conservation policies, from Aichi Target 11 to Target 3 of the Kunming–Montreal GBF, which calls for “well-connected” systems of protected areas [30,31], despite only about half of the land under protection being effectively connected [32]. Because a new PA’s contribution to whole-conservation-network connectivity depends on its topological position, it may fill a gap, bridge a corridor, or merely add an isolated node, and it is therefore essential to consider how suitable sites should be selected to maximise the overall connectivity of existing PA networks.

These three dimensions of the PA siting problem, namely, where to protect, for what objectives, and how to connect, have rarely been addressed within a single prioritisation framework. Xinjiang is the largest province of China, and retains expansive, low-pressure wilderness and globally significant arid and mountain biodiversity, yet its existing PA network under-represents key species and ecosystems [33]. In recent decades, agriculture, mining, energy and transport infrastructure have expanded rapidly in Xinjiang [34,35], which has fragmented the landscapes that a wide range of species depend on. In this study, we mapped priority areas to optimise PA networks in Xinjiang using systematic conservation planning. We formulated four conservation objectives and identified priority areas for each through minimum-set optimisation, using a human-pressure cost layer and a boundary penalty to minimise fragmentation. We then quantified the spatial mismatch among the priority areas of the four objectives and their conservation gap relative to the existing PA network, assessed the coverage of key species within the priority areas and existing PAs, and used graph-theoretic connectivity analysis to rank sites by their contribution to network connectivity.

## 2. Materials and Methods

### 2.1. Study Area

**Figure 1:**
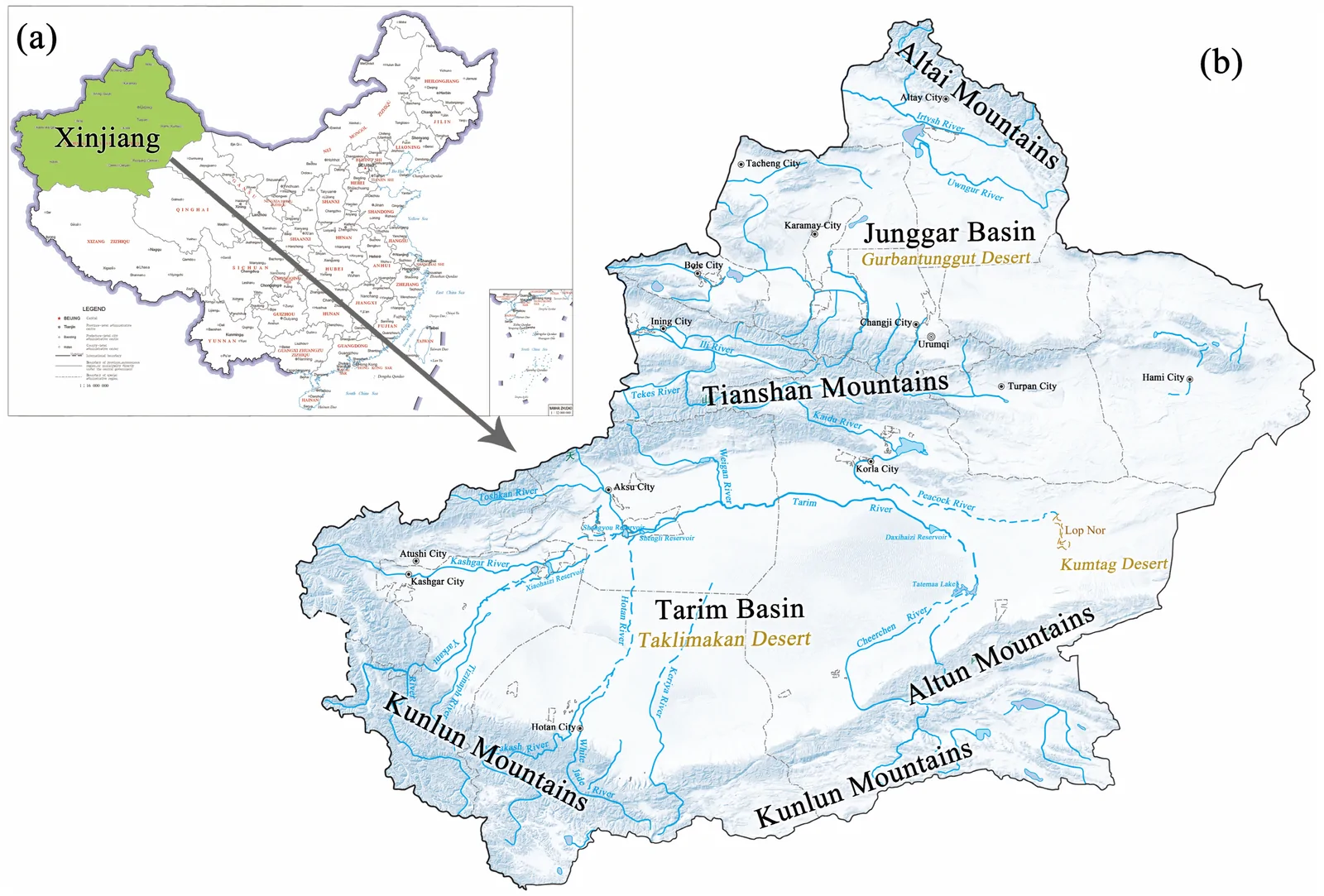
Location and topography of Xinjiang, China. Sources: (**a**) Standard Map Service of the Ministry of Natural Resources of China (map approval number: GS (2019)1686); (**b**) Department of Natural Resources of Xinjiang Uygur Autonomous Region (map approval number: Xin S (2023)063).

Our study was carried out in Xinjiang, northwest China (Figure 1), covering an area of 1.66 × 10^6^ km^2^. The topography of Xinjiang consists of alternating mountains and basins from the north to south, including the Altai Mountains, Junggar Basin, Tianshan Mountains, Tarim Basin, and Kunlun Mountains. The Taklamakan Desert is located in the middle of the Tarim Basin, covering an area of approximately 3.3 × 10^5^ km^2^, and is the largest desert in China. Xinjiang is situated within a conventional temperate continental climatic region, characterised by notable diurnal temperature fluctuations, limited precipitation, and significant evaporation rates. The effects of climate change, land-use change, rapid urbanisation, and ongoing economic development have collectively given rise to a spectrum of ecological challenges in Xinjiang, such as the degradation of grasslands and wetlands, loss of biodiversity, salinisation, and desertification of soil [36]. Xinjiang contains a wide variety of biological resources and unique species, including more than 730 vertebrate species, of which 178 are listed for state-level key protection, such as the Przewalski’s horse (*Equus ferus*), kiang (*Equus kiang*), Tibetan antelope (*Pantholops hodgsonii*), and snow leopard (*Panthera uncia*).

### 2.2. Processing the PA Data

We compiled the best available PA boundary data for Xinjiang from the National Forestry and Grassland Administration (https://www.forestry.gov.cn/, accessed 12 May 2023), the Key Biodiversity Areas [37] (https://www.keybiodiversityareas.org/, accessed 10 June 2023), and The World Database on Protected Areas [38] (www.protectedplanet.net, accessed 20 June 2023). When combined, these multiple sources yielded 160 PAs with a total area of approximately 2.24 × 10^5^ km^2^, covering 13.49% of Xinjiang’s land surface.

### 2.3. Processing the Conservation Objectives and Cost Data

We set four conservation objectives: biodiversity, landscape connectivity, ecosystem services, and an integrated objective. In addition, two cross-cutting targets were applied to all four objectives: rarity-based graded targets for six natural ecosystem types, and a 30% total-area target aligned with the 30 × 30 goal of the Kunming–Montreal Global Biodiversity Framework.

Biodiversity objective: Biodiversity was represented at the species level and used the individual feature of Area of Habitat (AOH) for each species, from plants, amphibians, reptiles, mammals, and birds. AOHs of the four former taxonomic groups were calculated following Brooks et al. [39], by intersecting distribution polygons obtained from the IUCN Red List, and were restricted to Existing, Native, and Resident status [40], determined by the intersection of IUCN habitat types and species elevational ranges (https://www.iucnredlist.org/en), digital elevation model (30 × 30 m resolution) and ESA CCI land cover (300 × 300 m resolution) [41], computed at 300 m and aggregated to a 1 km equal-area grid. AOHs of birds (1 × 1 km resolution) were downloaded from BirdLife International [42]; a systematic habitat-consistency correction was also applied. Only species with both a distribution polygon intersecting Xinjiang and an assigned IUCN Red List category (excluding Not Evaluated and Data-Deficient) were considered; finally, 591 species had AOH within Xinjiang (247 mammals, reptiles, amphibians, and plants; 344 birds). Conservation targets followed an area- and threat-based scheme [43,44]: threatened species (CR/EN/VU) were assigned a target of 100% where AOH < 2000 km^2^, 2000 km^2^ where AOH ranged from 2000 to 10,000 km^2^, and 20% of AOH where AOH > 10,000 km^2^; non-threatened species were assigned 10% of AOH.

Landscape connectivity objective: Landscape connectivity was derived from the aforementioned ESA CCI land-cover map, with three dispersal distances (10, 30 and 100 km) used to generate current-density maps, which covered the median dispersal abilities of most terrestrial species [28]. Each land-cover class was assigned a resistance value ranging from 1 to 500 to reflect the relative difficulty of movement, following the expert-opinion approach [45]: water and forest = 1; shrubland, grassland and lichen/moss = 10; wetland = 20; mosaics = 30; sparse vegetation = 50; bare ground and snow/ice = 100; cropland = 200; and urban areas = 500. Landscape connectivity was then quantified using omnidirectional circuit-theoretic modelling (Omniscape) [46]. This feature was assigned a 30% target of the total feature value.

Ecosystem services objective: Ecosystem services were represented by four indicators: soil retention, windbreak and sand fixation, water yield, and net primary productivity (NPP, a proxy for carbon sequestration). These were obtained from a modelled, nationally calibrated ecosystem-services dataset for China in 2020 (30 × 30 m resolution) [47], which integrates high-resolution land-cover data (GlobeLand30), multi-source remote sensing (Landsat for vegetation indices; MODIS for evapotranspiration and snow cover), and meteorological and soil data, all harmonised to 30 m resolution in an Albers equal-area projection. The four indicators were simulated with locally calibrated models: NPP with the Carnegie–Ames–Stanford Approach, soil retention with the Revised Universal Soil Loss Equation, windbreak and sand fixation with the Revised Wind Erosion Equation, and water yield with the InVEST water-yield module. These four indicators were each assigned a 30% target of the total feature value.

Integrated objective: The integrated objective combined all features from the three former objectives, 591 species-level AOH features, landscape connectivity, soil retention, windbreak and sand fixation, water yield, and net primary productivity, reflecting the biodiversity, ecosystem-function, and ecosystem-services dimensions that GBF Target 3 explicitly prioritises. Its target was defined as simultaneously meeting the targets of the three individual objectives.

The biodiversity and service capacity varied with ecosystems, and identifying priorities with a uniform threshold would systematically bias the prioritisation toward certain ecosystem types while undervaluing the conservation significance of others; we therefore stratified the prioritisation by ecosystem type. Natural ecosystems in Xinjiang were classified into nine types, forest, shrubland, grassland, desert, wetland, waterbody, cropland, urban areas, and glaciers, based on the aforementioned ESA CCI land-cover map. Ecosystem-type targets followed a rarity-based graded scheme [48,49]: wetland 80%, forest 60%, shrubland 50%, waterbody 40%, grassland 30%, and desert 20%. Cropland, urban areas, and glaciers were excluded from the ecosystem-type features because these targets represent natural ecosystems. This does not imply that such land lacks value: farmland-dependent birds are protected through their species-level AOH (cropland was listed as a suitable habitat in IUCN), and the water-provision value of glaciers is captured by the water-yield service feature.

We built a Regional Anthropogenic Pressure Index (RAPI) to quantify cumulative human land-use pressure and represent the cost of protection, which combined three components: grazing intensity, transport infrastructure, and resource extraction, as they were standard constituents of cumulative-pressure frameworks [14,50], and were the dominant, well-documented drivers of habitat degradation in the region [34,51,52]. Grazing intensity (0.0025° × 0.0025° resolution) in 2024 was obtained from the National Science & Technology Infrastructure of China [53]; transport infrastructure was calculated based on the railway and highway data in 2022, which were obtained from the National Catalogue Service for Geographic Information of China and the National Earth System Science Data Center; and resource extraction was quantified with distance decay exp(−d/10 km), with mining area data obtained from Maus et al. [54]. The pressures of cropland and urban areas were excluded because they were the complement of the natural-coverage feature in the conservation objectives, so their inclusion would double-count that signal. Each component was winsorised at its 99th percentile to limit the influence of extreme outliers (e.g., urban cores and mine clusters), and then they were min–max–normalised to 0–1, and averaged with equal weights.

### 2.4. Systematic Conservation Planning

All conservation objective and cost layers were standardised to a 1 km Behrmann equal-area projection (EPSG:54017) to ensure spatial consistency in area-based analyses.

We formulated and solved a minimum set covering problem using the R–4.6.1 package prioritizr 8.1.0 [55]: total cost (the sum of RAPI across selected planning units) was minimised subject to all feature targets being met, with binary decision variables and the HiGHS 1.14.0 exact solver (optimality gap ≤ 0.6%). To reduce the fragmentation of the resulting priority areas, a boundary penalty (1 × 10^−6^) was applied, penalising solutions by the total length of shared boundary between selected and unselected units. The problem was solved for each of the four objectives: biodiversity (BD), landscape connectivity (CONN), ecosystem services (ES), and the integrated objective combining all three (INTG). In addition, two scenarios were set, without existing protected areas (no PAs) and with existing protected areas locked in (with PAs; locked-in), yielding eight priority-area solutions in total (hereafter referred to by objective and scenario, e.g., BD–no PAs, CONN–with PAs). We did not assign explicit weights to features; instead, each feature was assigned a conservation target, and the minimum-set optimisation identified the minimum-cost set of planning units that simultaneously met all targets. Feature importance was therefore encoded by the magnitude of its target. We used 10 km planning units, which are appropriate for a province-scale analysis and computationally tractable.

### 2.5. Human Footprint (HFP) Three-Zone Classification

We used the 2024 human footprint index (HFP) [56] to characterise the intensity of cumulative human pressure across Xinjiang, and classify it into three zones following Williams et al. [57]: wilderness (HFP < 1), intact (1 ≤ HFP < 4), and modified (HFP ≥ 4). These three zones accounted for 47.3%, 22.7%, and 30.1% of Xinjiang, respectively. The three-zone classification was used to describe the human-pressure context of the priority areas (i.e., the proportion of each priority area falling within wilderness, intact, and modified zones), rather than as a conservation feature.

### 2.6. Coverage of Key Species Across Objectives

To assess the protective efficacy of priority areas identified by the four conservation objectives, we selected eight key species according to an overall consideration of taxonomic breadth, conservation status (Endangered, Vulnerable, and Near-threatened), and distribution range (wide-ranging or range-restricted). Finally, eight species were selected: snow leopard (VU), wild Bactrian camel (*Camelus ferus*, CR), Ili pika (*Ochotona iliensis*, EN; AOH 1034 km^2^), wild yak (*Bos mutus*, VU), Tibetan antelope (NT), white-headed duck (*Oxyura leucocephala*, EN), Central Asiatic frog (*Rana asiatica*, VU), and *Fritillaria cirrhosa* (VU; AOH 4.4 km^2^).

### 2.7. Quantifying Landscape Connectivity and the Contribution of Individual Patches

The probability of connectivity (*PC*) was used to assess the overall connectivity of the extant PAs and priority areas in Xinjiang, which is the probability that two individuals randomly placed in two patches can reach each other, estimated using the following Equation (1) [58]:

(1) $$\mathrm{PC}=(\sum_{i=1}^{n}\sum_{j=1}^{n}a_{i}\times a_{j}\times P_{\mathrm{ij}}^{*})/A_{L}^{2}\;$$

where *a_i_* and *a_j_* are the areas of patches *i* and *j*, respectively; *A_L_* is the area of Xinjiang; $P_{\mathrm{ij}}^{*}$ is the maximum product probability of all possible paths between patches *i* and *j*; and $P_{\mathrm{ij}}^{*}$ = 0 represents two patches that are completely isolated from each other. *PC* ranges from 0 to 1, with a higher *PC* value indicating better connectivity. The calculated method of $P_{\mathrm{ij}}^{*}$ followed Bunn et al. [59] and Herrera et al. [60].

The contribution of different patches to the overall connectivity (*dPC*) was calculated according to Saura and Pascual-Hortal [58], as follows in Equation (2):

*dPC(%) = (PC − PC^′^)/PC × 100%* (2)

where *PC* and *PC′* are the values of *PC* when an individual patch is present and removed from the studied landscape, respectively.

In accordance with individual-site connectivity, we also used 10, 30, and 100 km as median dispersal distances. The heterogeneity of land outside the extant PAs and identified priority areas was not considered.

All of these analyses were performed using Conefor Sensinode 2.2 [61].

### 2.8. Sensitivity Analysis

As conservation targets are partly policy-driven and inherently uncertain [62], we assessed the robustness of our conclusions to six sets of perturbations: (1) the species-level targets (the threshold AOH of threatened species were 1500 or 2500 km^2^); (2) the landscape-connectivity target (10–60%); (3) the ecosystem-services targets (10–60%); (4) the ecosystem-type graded targets (±10 percentage points); (5) the cost-layer component weights (each component doubled in turn) (Table S1); and (6) the boundary penalty (0 to 3 × 10^−6^) (Table S2). For each perturbation, the priority areas were resolved under the same minimum-set framework and compared with the baseline solution in terms of spatial extent and overlap, to verify that our conclusions were not artefacts of particular parameter choices. These perturbations left the priority-area extent essentially unchanged, confirming that the conclusions were not artefacts of particular parameter choices.

## 3. Results

### 3.1. Priority Areas Under No-PA Scenarios and Their Spatial Mismatch with Existing Protected Areas

Existing PAs cover only 13.49% of Xinjiang and fall short of nearly every conservation target: only 248 (41.96%) species meet their conservation targets; they capture just 19.94–23.13% of landscape connectivity and 3.50–13.62% of individual ecosystem services, indicating a large protection gap between existing PAs and the 30% target of the Kunming–Montreal Global Biodiversity Framework. Among ecosystem-type targets, only water bodies met their target (56.1% vs. 40%), and the rest failed, with shrubland and forest particularly deficient (8.88% vs. 50% and 27.95% vs. 60%, respectively). In contrast, for all four no-PA solutions, which selected approximately 30% of the region and met the targets of the features defining their respective objectives, as well as every ecosystem type. However, the three single-objective solutions did not satisfy the targets of the other objectives simultaneously; landscape connectivity and ecosystem services solutions only met 70.39% and 78.85% of species targets and landscape connectivity solutions only met 16.23–29.94% of ecosystem services targets, although all three solutions met the connectivity target (34.70–40.05%) (Table S3). Four solutions comprised 111–210 patches (ranged from 100 to 205,800 km^2^) and differed markedly in cost: the landscape connectivity solution was the least fragmented and cheapest, followed by ecosystem services, whereas the biodiversity and integrated solutions were the most fragmented and costly (Table S4).

**Figure 2:**
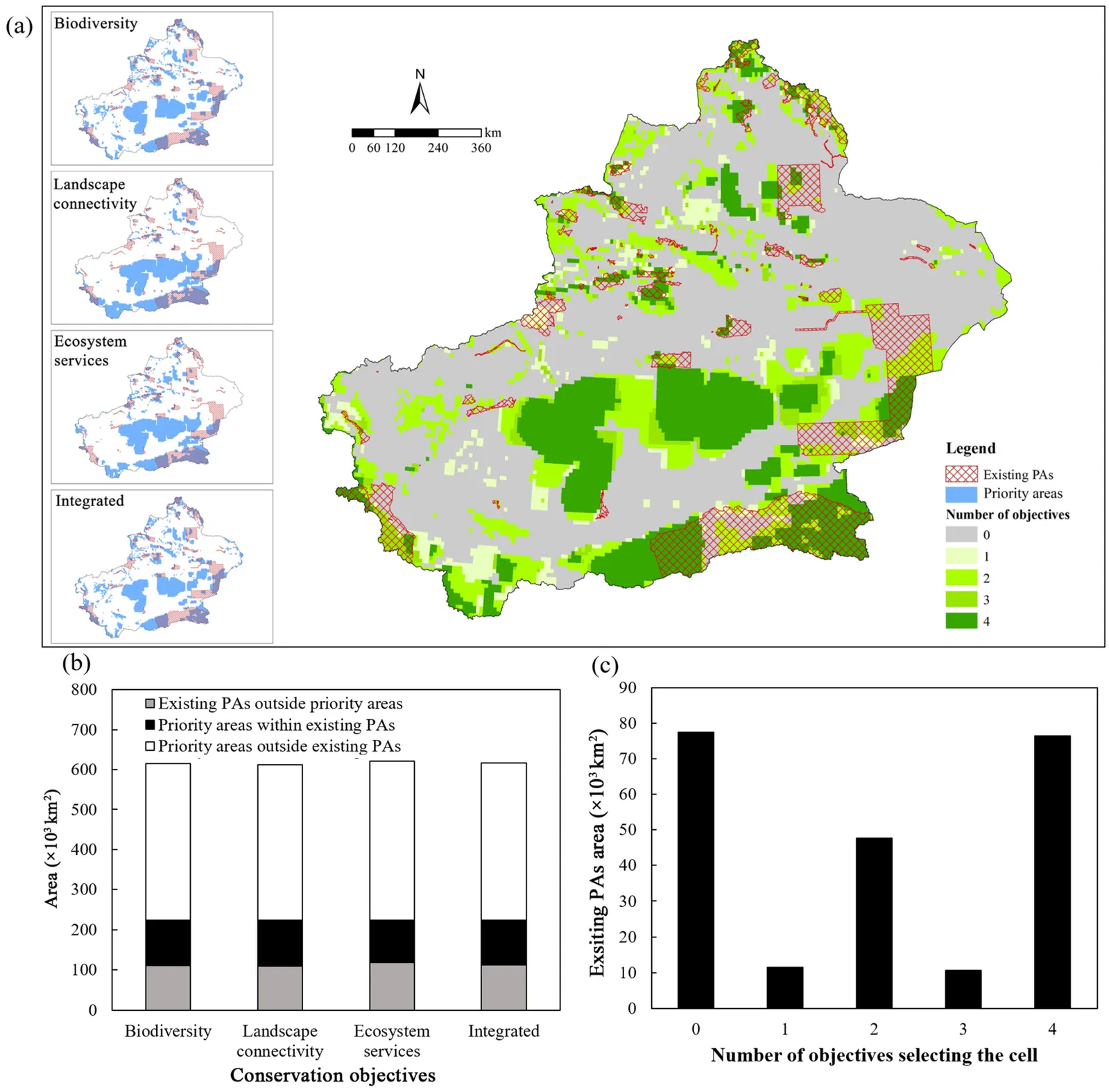
Priority areas under the no-PA scenarios and their spatial mismatch with existing PAs. (**a**) Priority areas identified for the four objectives and the consensus map of areas selected by zero-to-four objectives. (**b**) Area of existing PAs overlapping with priority areas for each objective. (**c**) Area of existing PAs located in regions selected by zero-to-four objectives.

The priority areas identified under the three single-objective solutions overlapped substantially, sharing 3.22 × 10^5^ km^2^, approximately 19.8% of Xinjiang’s total area. In general, all four no-PA solutions concentrated their priority areas in the south of Xinjiang, chiefly in the Tarim Basin and the Kunlun–Altun Mountains (Figure 2a). The priority areas identified by the biodiversity and integrated objectives showed similar spatial structure; both were dispersed, with moderate priority also in the Altai Mountains and the Junggar Basin, whereas the Tianshan Mountains received the least priority. In comparison, the landscape connectivity and ecosystem services solutions were mainly distributed in the southern region, and very little were in the Tianshan, Junggar, and Altai regions (Figure 2a).

The priority areas of no-PA scenarios showed a marked spatial mismatch with the existing PA network; for each conservation objective, only 47.10–51.25% of the existing PA area fell within the priority areas (Figure 2b); and at the same time, 34.62% (7.74 × 10^4^ km^2^) of the existing PA area was distributed in the regions selected by none of the four objectives, while 34.22% (6.98 × 10^4^ km^2^) was distributed in regions selected by all four objectives, and the remaining 31.34% (7.64 × 10^4^ km^2^) was in regions selected by one-to-three objectives (Figure 2c). In addition, 77.22–79.06% of the priority areas (about 3.9 × 10^5^ km^2^) was distributed outside existing PAs and therefore needs to be taken into the conservation network.

### 3.2. Complementing the Existing PA Network: New, Bridging, and Expansion Zones Under With-PA Scenarios

With existing PAs locked in, all of the priority areas identified by the four conservation objectives met the targets of the features. However, similar to the no-PA scenarios, the three single-objective solutions still fell short: the priority areas of the landscape connectivity objective and the ecosystem services objective only met 77.16% and 81.73% of the demand of species, respectively; the landscape connectivity objective only captured 19.23–26.16% of the ecosystem services requirement, and the biodiversity objective only satisfied 20.05% of windbreak and sand fixation (full results are shown in Table S1). In general, the fragment and cost of the four-objective solutions of with-PA scenarios were slightly higher than that of no-PA scenarios (Table S4), while the integrated solution became less fragmented (210 patches vs. 189 patches), but also more expensive (RAPI cost 256.92 vs. 209.59).

The priority areas outside existing PAs (hereafter additional priority areas) were likewise concentrated in the south of Xinjiang, with the most areas distributed in the Tarim Basin, followed by the Kunlun–Altun Mountains; the additional priority areas of the landscape connectivity objective were the most southerly, and that of the biodiversity and integrated objectives were most dispersed. The Tianshan Mountains again received the least priority under each objective.

**Figure 3:**
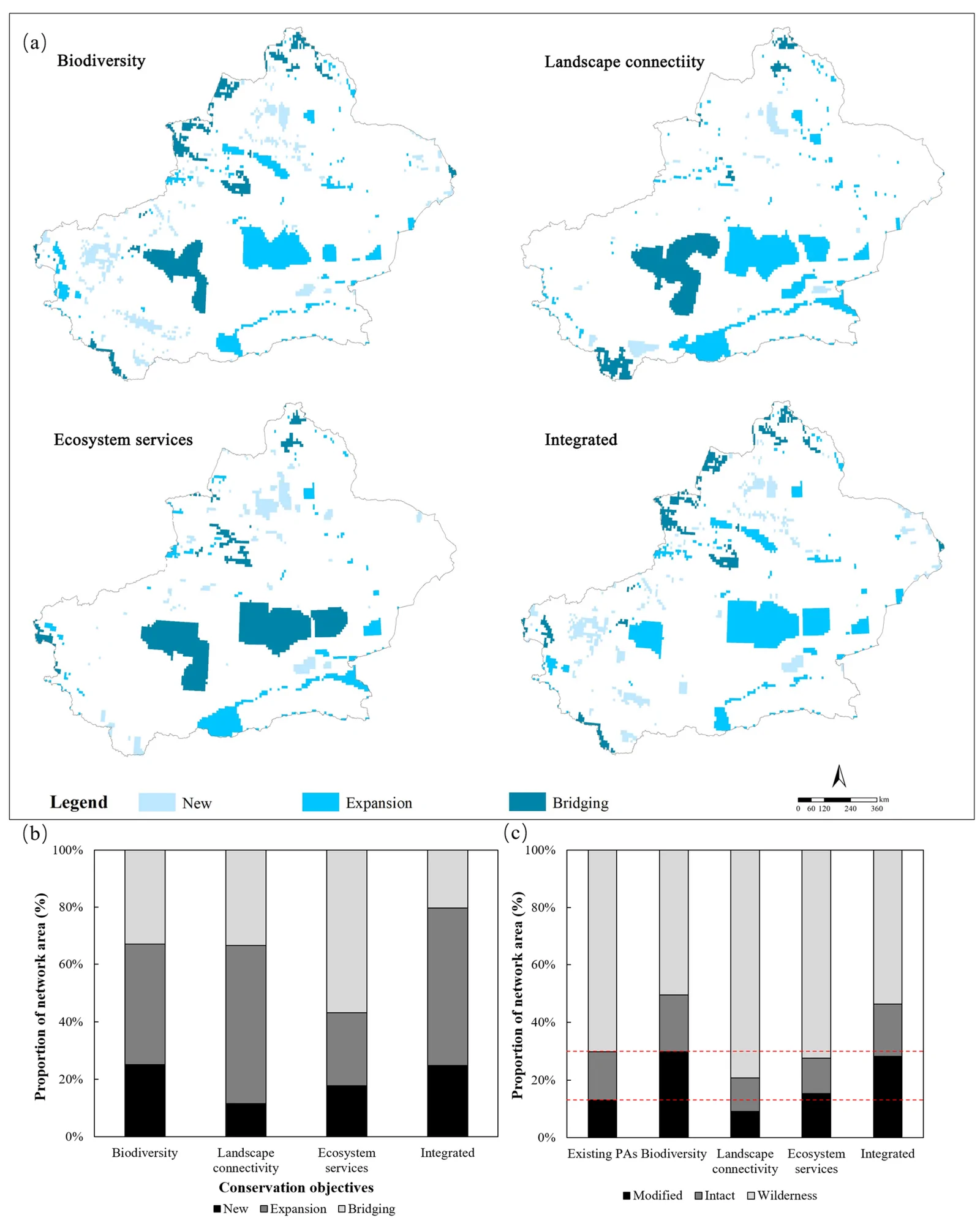
New, expansion, and bridging zones and human footprint composition of the additional priority areas under the with-PA scenarios. (**a**) The distribution of additional priority areas under the four objectives. (**b**) Area proportions of the new, expansion, and bridging zones of additional priority areas under the four objectives. (**c**) Human footprint three-zone composition (wilderness, intact, and modified) of the existing PAs and additional priority areas under the four objectives. Red dashed lines of (**c**) mark the boundaries of the existing PAs’ three zones, for comparison with the priority areas of the four objectives.

The additional priority areas were classed into three components, new, expansion, or bridging, according to the number of existing PAs (0, 1, or ≥2, respectively) that they adjoined. Expansion was the dominant zone for the biodiversity, landscape connectivity and integrated objectives, accounting for 41.94%, 55.09%, and 54.74% of the priority areas, respectively, whereas bridging dominated the ecosystem services solution (56.81%); new zones accounted for comparatively little across the four solutions (11.48–25.10%) (Figure 3b). The zones also differed in their patch structure: bridging zones consisted of relatively few patches (12–19), whereas new zones were formed by many small ones (67–122).

The proportions of existing PAs located in the wilderness, intact, and modified regions were 70.12%, 16.79%, and 13.09%, respectively, demonstrating that PAs have been preferentially established in areas of low human disturbance. By contrast, the additional priority areas extended into more disturbed regions: the biodiversity and integrated solutions placed 29.74% and 28.15% of their additional priority areas in modified regions, nearly double of the existing PAs, whereas the landscape connectivity solution tended to be more wilderness-biased (79.28% wilderness and only 9.16% modified), and the ecosystem services solution was intermediate (72.36% wilderness and 15.26% modified) (Figure 3c).

### 3.3. Species-Level Coverage of Key Species Across Objectives

Existing PAs in Xinjiang have limited capacity to protect key species, as they failed to meet the targets of four among the eight key species, especially the Ili pika (0.10% in existing PAs vs. 100% in target), and also the snow leopard (15.70% vs. 20%), Central Asiatic frog (18.12% vs. 20%), and white-headed duck (49.97% vs. 100%). Among the four solutions with PAs, the biodiversity and integrated solutions met the targets of all eight key species, while the landscape connectivity and ecosystem services solutions left these two range-restricted species almost entirely unprotected (Ili pika: 0.58% and 0.29%; white-headed duck: 51.6% and 53.9%, respectively), while the landscape connectivity and ecosystem services solutions left the Ili pika essentially unprotected (0.58% and 0.29% AOH coverage), and only covered half the habitat of the white-headed duck (51.56% and 53.88%), all far below the 100% targets (Table 1).

### 3.4. Connectivity Importance of Individual Priority Patches in the Conservation Networks

As single-objective networks were demonstrated to omit essential conservation targets of other objectives, we therefore restricted the connectivity-based prioritisation to the integrated objective. At the network level, the probability of connectivity (*PC*) increased with dispersal distance for all four networks, and was consistently highest for the landscape-connectivity–with-PA solution at 30 and 100 km (2.64% and 4.73%, respectively), with the integrated–with-PA solution ranking third (1.05%, 1.82% and 3.81% at three dispersal distances, respectively) (Table S5).

**Figure 4:**
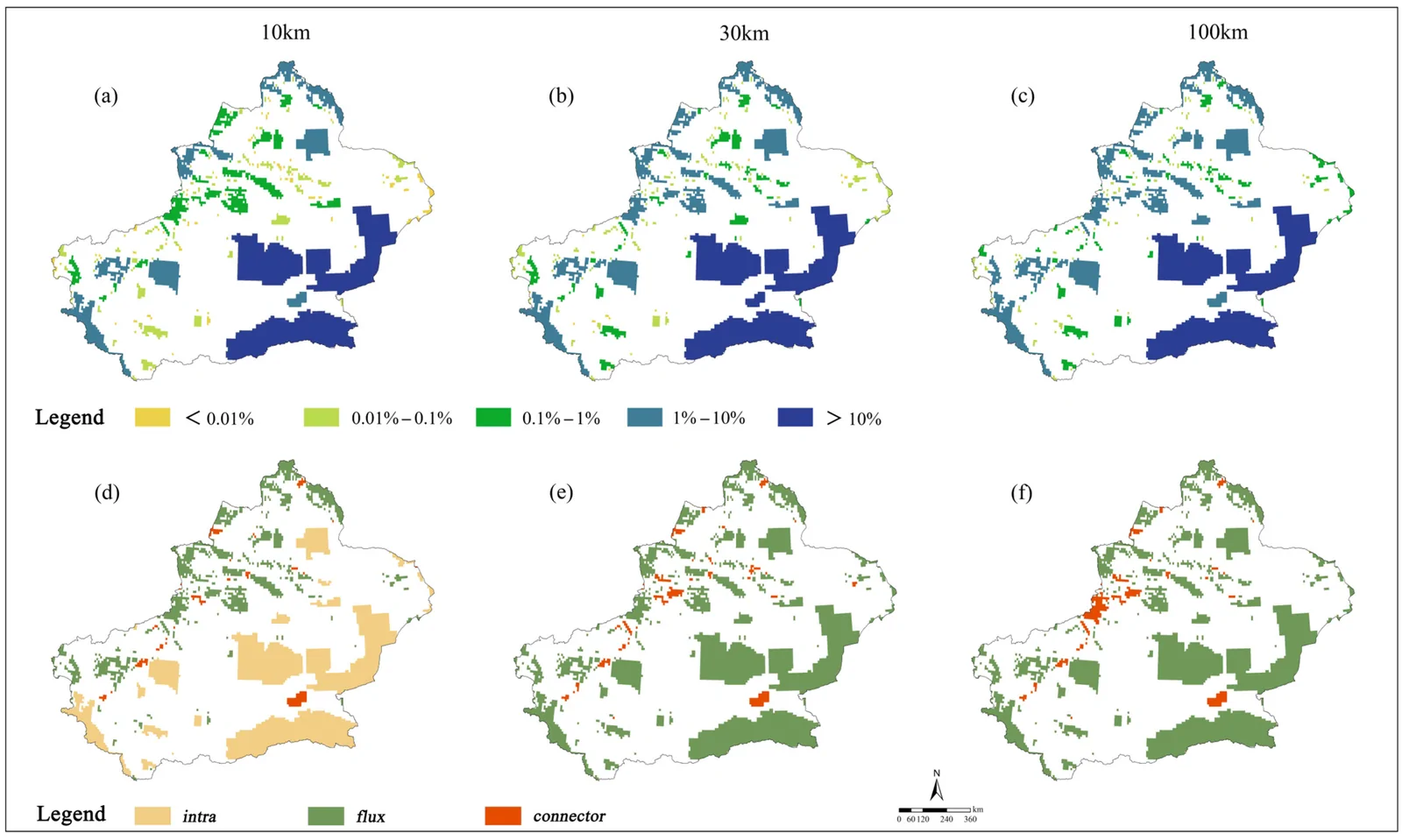
Connectivity importance of individual priority patches in the conservation network for integrated–with-PA solution. (**a**–**c**) Ranking of each priority patch that contributed to the overall connectivity, at 10, 30, and 100 km dispersal distances, respectively. (**d**–**f**) Dominant connectivity component (*intra*, *flux*, or *connector*) of each patch, at the same three dispersal distances.

The 189 patches of the integrated–with-PA solution were reclassified into five ranks based on their *dPC* values (< 0.01%, 0.01–0.1%, 0.1–1%, 1–10%, and >10%) (Figure 4). Across the three dispersal distances, only 3–4 patches attained the highest rank (>10% contribution; three at 10 km, four at 30 km, and three at 100 km); these large patches covered 2.48–2.54 × 105 km^2^, which was nearly half of the solution area, represent the principal connectivity providers and should therefore receive the highest conservation priority. In comparison, most of the smaller patches showed low contributions to maintaining overall landscape connectivity, with 56.08–81.48% of patches falling below 0.1% *dPC*. The dominant connectivity component also varied with dispersal distance: at 10 km dispersal distances, 19 patches were dominated by their own area (*intra*) (67.97% of solution areas), but none at 30 or 100 km; instead, flux emerged as the dominant component at 30 and 100 km. The connector component remained minor across all three dispersal distances, though its contribution showed an increasing tendency with distance (Figure 4). The role of area-driven importance shifted toward stepping-stone (*flux*) and *connector* as dispersal distances increased, underscoring the need to account for species’ dispersal abilities when creating new protected areas.

## 4. Discussion

### 4.1. Spatial Mismatch Among Priority Areas for Different Conservation Objectives and Existing PAs

Comprehensive knowledge of biodiversity patterns and ecological processes that further impact biodiversity is the basis for determining priority areas for mitigating biodiversity loss. Here, we identified priority areas for conservation based on different objectives, which is similar to the studies conducted in the Paraguayan Atlantic Forest [63], India [64], and global PAs [26], and found a spatial mismatch among the results for the individual objectives. The overlapping area of priority areas identified under three single-objective solutions was 3.22 × 10^5^ km^2^, which accounts for 19.76% of Xinjiang’s area. Relative to the 30% targets of each objective, this relatively high overlap suggests a shared, cost-driven convergence on the low-cost south: RAPI is much lower in the Tarim Basin and remote Kunlun–Altun Mountains than in northern Xinjiang, where population density and grazing pressure are higher; all three minimum-set solutions were drawn to the same southern region, leading to overlapping in roughly two-thirds of their area, with the remaining divergence arising mainly from the biodiversity solution extended across the whole region to capture range-restricted and scattered species; the connectivity solution reached toward the Altai and Tianshan mountains for habitat corridors; and ecosystem services solution reached partly toward desert margins through sand fixation, and partly toward northern regions for better vegetation and water. This cost-driven pattern is particularly evident in the species-rich Tianshan Mountains, which received surprisingly little priority. This may be explained by the predominance of widespread species in the regional pool (whose targets can be met in cheaper southern region), so that higher-cost, species-rich areas were retained only where required by range-restricted species.

Our study revealed that only 47.10–51.25% of the existing PA area fell within the priority areas, and 77.22–79.06% of priority areas (about 3.9 × 10^5^ km^2^) were distributed outside of the existing PAs, suggesting that a substantial share of conservation hotspots are currently unprotected and highlighting a considerable opportunity to prioritise future protection. This surprising result reflects the opportunistic manner in which most PAs were established. Existing PAs tend to be biased toward land of low economic value, such as high elevations, steep slopes, and extensive desert and rock-and-ice areas, rather than toward areas of high conservation importance [9,10]. This pattern is evident in Xinjiang: 70.12% of the existing PAs was located in wilderness, and only 13.09% was in modified landscapes, confirming that designation preferred areas of minimal human disturbance. They were also seldom designed to meet quantitative, representative targets for the multiple complements of species and habitat types that our systematic planning framework sought. This interpretation is supported by the low achievement rates of the existing PA network for most ecosystem types (e.g., shrubland 8.88%, grassland 15.95%, forest 27.95%), connectivity (19.94–23.12%) and ecosystem services (3.50–13.62%). Our species-level validation further underscored this shortfall. Existing PAs failed to meet the targets of four of eight key species examined, most strikingly the range-restricted Ili pika, which only had 0.10% of its AOH located in existing PAs, and to our knowledge, no PAs have been designated specifically for this species.

### 4.2. Optimisation of the Existing PA Network

To optimise Xinjiang’s existing PA network, we identified additional priority areas for new PAs under four objectives. As none of the single-objective solutions simultaneously met the targets of the others, efforts based on a single objective would not deliver effective biodiversity conservation, which highlights the importance of incorporating multiple objectives in the conservation decision-making processes. The spatial relationship of these additional areas to the existing PAs further indicates that the protection strategy should be sequenced in practice. Expansion and bridging zones dominated the biodiversity, connectivity and integrated solutions (74.90–88.52%), indicating that the most efficient route to the 30% target is to enlarge the existing PAs rather than to build multiple new small PAs. Combined with the result of *dPC*-based ranking, a tiered pathway was proposed: expand existing PAs first, secure high-connectivity bridges next, and refine the small new patches through fine-scale surveys last.

In general, wilderness and intact regions have a lower human population and less agriculture and industry, and, thus, less human–wildlife conflict than in the case of biodiversity protection in modified regions affected by human activities, such as clearing, farming, grazing, and mining. As a result, wilderness and intact regions could offer easier and cheaper opportunities for creating new PAs [65]. This asymmetry explained why our additional priority areas were concentrated in the less disturbed south across all four objectives: such regions offer the cheapest pathway toward the 30 × 30 target. However, about 30% of the additional priority areas under the biodiversity and integrated objectives were located in modified regions, or, in other words, some range-restricted species were concentrated in there. Therefore, bold conservation agendas will require the expansion of PAs or OECMs in human-dominated landscapes [20]. The IUCN management categories offer a framework for this differentiation: strict reserves (Category Ib) suit the near-pristine desert and mountain blocks of the south, whereas biodiversity priorities embedded in agricultural mosaics are better met by a less restrictive way, such as protected landscapes (Category V) and protected areas with sustainable use of natural resources (Category VI), which accommodate continued human use and maintain buffering and corridor functions [11]. The white-headed duck illustrates this distinction: its lakeshore and wetland habitats lie adjacent to intensively used lands, where strict exclusion is impractical, and OECMs’ governance types may be the only viable pathway. Therefore, a realistic route was to establish strict PAs in less human-disturbed areas and use Category V/VI instruments and OECMs (e.g., sanctuaries) across the oasis belts.

An effective approach to conserving biodiversity is the restoration and rewilding of additional priority areas in human-dominated landscapes [66], which can enhance a variety of ecosystem services [67], carbon sequestration [68], species diversity, and PA connectivity [69]. Such conservation action has been conducted in some places in Xinjiang, such as the Bogda World Natural Heritage Site and Houxia Town in Urumqi. However, large-scale restoration and rewilding have high costs, and Xinjiang is a less developed region of China, so conducting such activities without financial support from developed regions is not only unjust but also impossible. Regional cost-sharing and fiscal transfer mechanisms would, therefore, improve the efficiency of biodiversity conservation in China. In some circumstances, restoration and rewilding activities involve the displacement or relocation of people; however, indigenous peoples and local communities have demonstrated their effectiveness as custodians of biodiversity on a global scale, as evidenced by numerous cases [9]. Therefore, these sites are not appropriate for establishing strict PAs. Reinforcing the capacity of indigenous peoples and local communities to participate in conservation provides a primary pathway for safeguarding biodiversity.

Several additional actions can also promote biodiversity conservation outcomes during the optimisation of existing PA networks. Strict protection might not be appropriate or feasible for all land; therefore, in priority conservation areas, the expansion of roads, mining, and transport infrastructure should ensure “no net loss” of biodiversity and natural ecosystems [70], and the scale of such developments should be limited or gradually ceased. In addition, limiting further modifications and reducing the human footprint would reduce PA isolation, which is critical for the long-term persistence of species when facing the pressures of land-use and climate change [71]. Furthermore, innovative conservation solutions, such as conservation easements, payments for ecosystem services, community-based conservation, and working land conservation, are necessary in heavily modified regions [72].

### 4.3. Optimised PA Network and the Role of Individual Patches

Habitat fragmentation is a major threat to biodiversity. Our results show that the overall landscape connectivity of existing PAs in Xinjiang is low, indicating a limited capacity to resist the stress being placed on biodiversity by climate change and human disturbance. We also found that including more sites in the PA network can increase overall landscape connectivity.

The resources available for PA establishment and management are limited. Therefore, to maximise conservation outcomes with limited resources, ranking priority areas is necessary. We ranked the patches of the integrated solution based on their contribution to overall landscape connectivity (*dPC*) and found that larger patches made higher contributions: only three-to-four patches (covering nearly half of the solution area) contributed more than 10% of connectivity, whereas 56.08–81.48% of patches fell below 0.1%. Indeed, the importance of large patches in fragmented landscapes has been acknowledged globally, and apart from playing the role of a connection provider, larger patches also support more species and larger populations and provide better interior habitats [73]. Therefore, conservation efforts have typically focused on large patches, which should be prioritised when establishing new PAs [1,24].

Nevertheless, small patches should not be ignored as they can act as stepping stones to promote the long-distance dispersal of species, and deliver greater ecosystem services and harbour more species than a few large patches of the same total area [61,74]. Our component decomposition showed that, as dispersal distance increased, the dominant role of patches shifted from their own area (*intra*) toward stepping-stone (*flux*) and *connector* functions, underscoring the need to account for species’ dispersal abilities when creating new PAs. The required area for small patches also varied with functions: for range-restricted species, it must be sufficient to sustain a viable population [75], whereas for patches serving chiefly as stepping stones for connectivity, a few square kilometres or even less may suffice. However, in highly modified environments, the low costs of restoration and rewilding of small patches make them more likely to be implemented in the short term. Therefore, we suggest that conservation efforts should focus on large patches in the wilderness and intact regions of Xinjiang and pay more attention to small patches in the modified regions.

### 4.4. Study Limitations

Three limitations should be noted. First, our species dataset was restricted to terrestrial vertebrates and plants with available range maps; invertebrates, fungi, species occurring in Xinjiang but lacking an IUCN range map (e.g., the Horsfield’s tortoise, *Testudo horsfieldii*), and reintroduced species such as Przewalski’s horse were likewise omitted. Second, the biodiversity data was the AOH of species, which identify where a species is likely to persist on the basis of habitat suitability, but treat all suitable habitats as equivalent; areas supporting high-density populations may consequently be under-weighted. Third, the input data span 2019–2026 rather than a single year: the ecosystem services and cost estimates represent early-2020s conditions, the species AOH may overestimate current habitat owing to post-2022 land-cover change, and the mine footprint may underestimate pressure along energy-development corridors. This inconsistency is unlikely to alter the configuration of the priority areas, as rapidly changing land covers are largely excluded from the networks (cropland and urban land account for only 1.24–7.55% of the priority areas). Future work should broaden the taxonomic scope to include under-represented groups, incorporate abundance or population-density data, and periodically update the priority areas as newer data layers become available. In particular, abundance-based targets would direct protection toward the areas most important for sustaining viable populations rather than suitable habitats alone.

## 5. Conclusions

We explored the spatial overlap and mismatch of priority areas for conservation in Xinjiang, China, and highlighted the importance of using multiple objectives in area-based conservation actions. In addition, our results demonstrate that more than 70% of the priority areas in Xinjiang remain unprotected, and many existing PAs are not in the ideal locations. To address this, we identified priority areas outside of existing PAs to improve biodiversity conservation and explored multiple strategies to optimise the PA network, namely establishing new PAs, restoring or rewilding conservation hotspots, participation of indigenous people in conservation in human-dominant landscapes, reducing the human footprint, regional cost-sharing, and fiscal transfer. Finally, our results confirm that protecting more sites could increase the connectivity of PAs in this region. Based on the contribution of individual patches to overall landscape connectivity and the associated economic costs, we suggest a dual strategy of protecting large patches in wilderness and intact regions alongside the restoration of small patches in modified regions. Future biodiversity conservation work should consider land-use policies and balance conservation costs with protection efficiency.

## Figures and Tables

**Table 1:** Coverage of key species under existing protected areas and four conservation objectives (with-PA scenarios).

Species	Target	Existing PAs	BD–with PAs	CONN–with PAs	ES–with PAs	INTG–with PAs
Tibetan antelope	10%	65.29%	73.69%	89.72%	75.09%	73.17%
Snow leopard	20%	15.70%	32.69%	30.49%	29.25%	31.55%
Wild Bactrian camel	20%	26.93%	34.58%	45.79%	36.59%	34.04%
Wild yak	20%	60.99%	74.46%	90.93%	89.18%	71.86%
Central Asiatic frog	20%	18.12%	35.12%	28.84%	45.58%	35.65%
White-headed duck	100%	49.97%	100%	51.56%	53.88%	100%
Ili pika	100%	0.10%	100%	0.58%	0.29%	100%
*Fritillaria cirrhosa*	100%	100%	100%	100%	100%	100%

**Note:** BD: biodiversity, CONN: landscape connectivity, ES: ecosystem services, INTG: integrated.

## Data Availability

The data presented in this study are available on request from the author; the data are also part of an ongoing study.

## Supplementary Materials

The following supporting information can be downloaded at https://www.mdpi.com/article/10.3390/biology15201863/s1, Table S1: Sensitivity of the priority-area solutions to alternative target settings and cost-layer component weights; Table S2: Sensitivity of priority-area solutions to the boundary penalty (0 to 3 × 10^−6^); Table S3: The achievement rate of protection element targets (%); Table S4: Boundary length, number of patches, and RAPI cost of the eight priority areas under the no-PA and with-PA scenarios; Table S5: Overall landscape connectivity of priority patches for different conservation objectives with three selected dispersal distances.

## Abbreviations

The following abbreviations are used in this manuscript:

PA	Protected area
GBF	Global Biodiversity Framework
CBD	Convention on Biological Diversity
OECM	Other effective area-based conservation measure
DEM	Digital elevation model
*PC*	Probability of connectivity
*BD*	Biodiversity
*CONN*	Landscape connectivity
*ES*	Ecosystem services
*INTG*	Integrated
