# Supplementary material for: Multi-Objective Spatial Prioritisation for Protected-Area Network Expansion in Xinjiang, China
Source: Biology (Basel). 2026 Oct 16;15(20):1863. doi: 10.3390/biology15201863 (PMC13701901; doi:10.3390/biology15201863)
Supplement: Supplementary file 1 [file biology-15-01863-s001.zip › biology-4536249-supplementary.pdf]

Supplementary Materials: Sensitivity analysis, target achievement, and spatial configuration of Priority Areas

Across all perturbations, objectives and scenarios, the priority areas remained broadly stable (Jaccard similarity 0.47–1.00), and the selected area stayed near 30% of the region except when representation targets were raised. The greatest sensitivity emerged under elevated targets: the connectivity solution expanded to ~54% of the region at a 60% target, and the ecosystem-service solution rearranged substantially (Jaccard 0.47-0.69 at 50-60% targets), reflecting the greater difficulty of meeting higher targets under a minimum-set formulation. In contrast, the threat bonus (Jaccard 0.80-0.93), the ecosystem-type graded targets (0.69-0.88) and the cost-layer weights (0.76-0.92) induced only modest changes, and relaxing the connectivity target under the with-PA scenario left the solution unchanged (Jaccard = 1.00). Results were consistent between the with- and without-existing-PA scenarios, confirming that our conclusions are robust to reasonable uncertainty in parameter values.

Table S1. Sensitivity of the priority-area solutions to alternative target settings and cost-layer component weights.

Perturbation	Objective	Value	Priority areas proportion (%)	Cost	Jaccard
Threat bonus	Biodiversity-no PAs	1.5	30.24	199.4	0.7958
	Biodiversity-with PAs	1.5	30	253.5	0.9347
	Integrated-no PAs	1.5	30.03	195	0.8446
	Integrated-with PAs	1.5	30	251.1	0.8389
	Biodiversity-no PAs	2.5	30.01	213.3	0.8426
	Biodiversity-with PAs	2.5	31.19	259	0.8194
	Integrated-no PAs	2.5	30.04	213.5	0.8374
	Integrated-with PAs	2.5	31.37	259.3	0.8696
Landscape connectivity targets	Landscape connectivity-no PAs	0.1	30	81.4	0.9728

	Landscape connectivity-with PAs	0.1	30	134.5	1
	Integrated-no PAs	0.1	30.06	209.6	0.9503
	Integrated-with PAs	0.1	30	256.9	1
	Landscape connectivity-no PAs	0.2	30.01	81.3	0.9772
	Landscape connectivity-with PAs	0.2	30	134.5	1
	Integrated-no PAs	0.2	30	209.5	0.926
	Integrated-with PAs	0.2	30	256.9	1
	Landscape connectivity-no PAs	0.4	31.35	85.5	0.8221
	Landscape connectivity-with PAs	0.4	31.35	135	0.943
	Integrated-no PAs	0.4	30.01	209.9	0.9112
	Integrated-with PAs	0.4	30	257.5	0.9391
	Landscape connectivity-no PAs	0.5	44.97	137.1	0.655
	Landscape connectivity-with PAs	0.5	45.07	168.5	0.6443
	Integrated-no PAs	0.5	44.36	236.2	0.6385
	Integrated-with PAs	0.5	44.58	273.1	0.6604
	Landscape connectivity-no PAs	0.6	53.44	216.5	0.5545
	Landscape connectivity-with PAs	0.6	54.03	241.8	0.5428
	Integrated-no PAs	0.6	52.74	299.3	0.5444
	Integrated-with PAs	0.6	53.62	321.5	0.5473
Ecosystem services targets	Ecosystem services-no PAs	0.1	30	81.3	0.7901
	Ecosystem services-with PAs	0.1	30	135.2	0.7722
	Integrated-no PAs	0.1	30.01	209.6	0.9281
	Integrated-with PAs	0.1	30	269.4	0.8219
	Ecosystem services-no PAs	0.2	30	81.5	0.8255
	Ecosystem services-with PAs	0.2	30	134.3	0.7712
	Integrated-no PAs	0.2	30.02	209.7	0.9214
	Integrated-with PAs	0.2	30	269.4	0.8219
	Ecosystem services-no PAs	0.4	30.01	140.6	0.7943
	Ecosystem services-with PAs	0.4	30	193.1	0.7595

	Integrated-no PAs	0.4	30.01	225	0.7892
	Integrated-with PAs	0.4	30.08	269.1	0.8546
	Ecosystem services-no PAs	0.5	30	206.5	0.5958
	Ecosystem services-with PAs	0.5	33.45	252.6	0.6916
	Integrated-no PAs	0.5	30	268.7	0.6247
	Integrated-with PAs	0.5	35.49	312.5	0.7698
	Ecosystem services-no PAs	0.6	30	281.2	0.4654
	Ecosystem services-with PAs	0.6	38.76	320.1	0.6378
	Integrated-no PAs	0.6	33.09	340	0.5163
	Integrated-with PAs	0.6	42.21	378.9	0.6435
Ecosystem type	Biodiversity-no PAs	low	30.01	195.5	0.7669
	Biodiversity-with PAs	low	30.24	257.5	0.8121
	Landscape connectivity-no PAs	low	30	51.5	0.7029
	Landscape connectivity-with PAs	low	30	112.6	0.7307
	Ecosystem services-no PAs	low	30.01	87.8	0.6902
	Ecosystem services-with PAs	low	30.01	142	0.8251
	Integrated-no PAs	low	30.02	197.3	0.8013
	Integrated-with PAs	low	30.12	250.9	0.8803
	Biodiversity-no PAs	high	33.21	239.9	0.7126
	Biodiversity-with PAs	high	33.58	278	0.7504
	Landscape connectivity-no PAs	high	31.65	132.4	0.709
	Landscape connectivity-with PAs	high	31.99	176	0.7177
	Ecosystem services-no PAs	high	32.15	134.4	0.7077
	Ecosystem services-with PAs	high	32.59	176.7	0.7233
	Integrated-no PAs	high	33.27	240.5	0.7331
	Integrated-with PAs	high	33.49	277.7	0.8001
Cost weight	Biodiversity-no PAs	grazing2x	30.08	393.8	0.7999
	Biodiversity-with PAs	grazing2x	30	491.9	0.8672
	Landscape connectivity-no PAs	grazing2x	30	142.5	0.881

Landscape connectivity-with PAs	grazing2x	30	243.7	0.8847
Ecosystem services-no PAs	grazing2x	30	192.6	0.775
Ecosystem services-with PAs	grazing2x	30	278.8	0.8426
Integrated-no PAs	grazing2x	30.04	387.6	0.8511
Integrated-with PAs	grazing2x	30	486.1	0.8517
Biodiversity-no PAs	transport2x	30.01	377.3	0.8105
Biodiversity-with PAs	transport2x	30.08	497.9	0.7895
Landscape connectivity-no PAs	transport2x	30.01	122.5	0.7845
Landscape connectivity-with PAs	transport2x	30.01	244.7	0.8274
Ecosystem services-no PAs	transport2x	30	167.4	0.7592
Ecosystem services-with PAs	transport2x	30	290.9	0.7876
Integrated-no PAs	transport2x	30.05	377.1	0.8205
Integrated-with PAs	transport2x	30	502.6	0.8375
Biodiversity-no PAs	mining2x	30.01	277	0.875
Biodiversity-with PAs	mining2x	30.04	360.9	0.8693
Landscape connectivity-no PAs	mining2x	30	97.4	0.8472
Landscape connectivity-with PAs	mining2x	30	175.2	0.861
Ecosystem services-no PAs	mining2x	30	127.1	0.8189
Ecosystem services-with PAs	mining2x	30.01	201.6	0.9157
Integrated-no PAs	mining2x	30.05	276.7	0.8627
Integrated-with PAs	mining2x	30	359.5	0.8497

The boundary penalty governed the compactness of the priority areas rather than their total extent or conservation content. Without a boundary penalty (penalty = 0), the solutions became markedly more fragmented (247-335 patches) and slightly larger (33.76-39.29% of the region), with Jaccard similarities of 0.65-0.70 relative to the baseline. Increasing the penalty to 2×10^{-6} or 3×10^{-6} produced solutions close to the baseline (Jaccard 0.74-0.88), while further reducing boundary length, although the biodiversity objectives sometimes failed to converge within the time limit at 3×10^{-6}. These results confirm that our conclusions are insensitive

to the exact value of the boundary penalty, which affects only the compactness of the resulting priority areas.

Table S2. Sensitivity of priority-area solutions to the boundary penalty (0 to 3 × 10^{-6}).

Objective	Penalty	Time (s)	Priority areas proportion (%)	Cost	Boundary length (km)	Jaccard
Biodiversity-no PAs	0	8.4	37.55	203.97	70580	0.654
Biodiversity-no PAs	1 × 10^{-6}	NA	30.01	209.65	39680	1
Biodiversity-no PAs	2 × 10^{-6}	153.9	30	218.9	37420	0.7881
Biodiversity-no PAs	3 × 10^{-6}	242.3	30.04	225.39	33420	0.7448
Biodiversity-with PAs	0	8.1	39.29	255.1	58540	0.6636
Biodiversity-with PAs	1 × 10^{-6}	NA	30	269.3	38440	1
Biodiversity-with PAs	2 × 10^{-6}	67.6	30	261.95	36820	0.8808
Biodiversity-with PAs	3 × 10^{-6}	88.5	30	266.73	36700	0.8193
Landscape connectivity- no PAs	0	0.9	33.76	72.11	58500	0.7017
Landscape connectivity- no PAs	1 × 10^{-6}	NA	30.02	81.25	25840	1
Landscape connectivity- no PAs	2 × 10^{-6}	57.8	30	89.79	19560	0.856
Landscape connectivity- no PAs	3 × 10^{-6}	240.7	30	98.6	20060	0.7514
Landscape connectivity- with PAs	0	1.1	37.65	127.51	51080	0.6936
Landscape connectivity- with PAs	1 × 10^{-6}	NA	30	134.47	28380	1
Landscape connectivity- with PAs	2 × 10^{-6}	57.4	30.05	137.01	21520	0.8187
Landscape connectivity- with PAs	3 × 10^{-6}	79.6	30.08	141.01	20080	0.7926
Ecosystem services-no PAs	0	0.9	35.47	91.13	65520	0.6817
Ecosystem services-no PAs	1 × 10^{-6}	NA	30	100.5	29440	1
Ecosystem services-no PAs	2 × 10^{-6}	93	30.01	110.58	22760	0.8272
Ecosystem services-no PAs	3 × 10^{-6}	169.4	30.02	118.81	21100	0.7396
Ecosystem services-with PAs	0	1	39.03	143.5	55880	0.6885
Ecosystem services-with PAs	1 × 10^{-6}	NA	30	147.41	28440	1

Ecosystem services-with PAs	2×10^{-6}	74.7	30	154.29	26080	0.872
Ecosystem services-with PAs	3×10^{-6}	94.2	30	161.17	21020	0.8101
Integrated-no PAs	0	4.5	37.55	203.97	70580	0.6544
Integrated-no PAs	1×10^{-6}	NA	30.05	209.59	39880	1
Integrated-no PAs	2×10^{-6}	183.5	30	218.76	35620	0.78
Integrated-no PAs	3×10^{-6}	242.6	30.22	244.04	39280	0.7419
Integrated-with PAs	0	4.5	39.29	255.1	58540	0.6814
Integrated-with PAs	1×10^{-6}	NA	30	256.92	36600	1
Integrated-with PAs	2×10^{-6}	73.6	30.27	263.25	38240	0.842
Integrated-with PAs	3×10^{-6}	108.3	30	266.9	37020	0.8245

Table S3. The achievement rate of protection element targets (%).

Protection elements	Targets	Existing PAs	BD-no PAs	CONN-no PAs	ES-no PAs	INTG-no PAs	BD-with PAs	CONN-with PAs	ES-with PAs	INTG-with PAs
Species	100%	41.96%	100%	70.39%	78.85%	100%	100%	77.16%	81.73%	100.00%
Landscape connectivity (10 km)	30%	23.13%	40.05%	38.12%	38.03%	40.05%	43.90%	40.80%	40.71%	43.11%
Landscape connectivity (30 km)	30%	22.34%	38.43%	36.59%	36.85%	38.42%	42.37%	39.36%	39.71%	41.56%
Landscape connectivity (50 km)	30%	19.94%	36.30%	34.70%	35.54%	36.33%	39.34%	36.52%	37.30%	38.55%
Net primary productivity	30%	11.24%	31.84%	19.23%	30.02%	31.85%	34.39%	20.98%	30.01%	33.45%
Windbreak and sand fixation	30%	3.50%	29.37%	29.94%	30.06%	31.02%	20.05%	24.39%	30.02%	30.12%
Water yield	30%	9.99%	31.49%	16.32%	30.02%	31.48%	34.89%	19.23%	30.02%	34.27%
Soil retention	30%	13.62%	33.74%	25.89%	30.08%	33.78%	36.23%	26.16%	30.99%	35.01%
Forest	60%	27.95%	60.08%	60.00%	60.00%	60.10%	60.85%	60.05%	60.03%	60.00%
Shrubland	50%	8.88%	50.00%	50.00%	50.00%	50.05%	50.00%	50.00%	50.07%	50.03%
Grassland	30%	15.95%	30.02%	30.00%	30.00%	30.00%	32.42%	30.05%	30.01%	30.91%
Desert	20%	12.70%	29.40%	31.85%	30.39%	29.48%	27.73%	31.38%	30.17%	28.44%
Wetland	80%	45.20%	80.01%	80.00%	80.00%	80.04%	80.54%	80.08%	80.02%	80.05%
Waterbody	40%	56.10%	57.81%	40.14%	40.20%	57.81%	79.51%	62.72%	62.96%	79.51%

Note: Red characters indicate that the target has not been met; BD = Biodiversity, CONN = Landscape connectivity, ES = Ecosystem services, INTG = Integrated.

Table S4. Boundary length, number of patches, and RAPI cost of the eight priority areas under the no PAs and with PAs scenarios.

Conservation objectives	Boundary length (km)	Patches				RAPI Cost
		Number	Minimum area (km^2)	Median area (km^2)	Maximum area (km^2)	
Biodiversity-no PAs	39680	204	100	400	66700	209.65
Landscape connectivity-no PAs	25840	111	100	200	205800	81.25
Ecosystem services-no PAs	29440	130	100	300	201000	100.50
Integrated-no PAs	39880	210	100	300	68000	209.59
Biodiversity-with PAs	38440	218	100	200	97200	269.30
Landscape connectivity-with PAs	28380	158	100	200	123400	134.47
Ecosystem services-with PAs	28440	138	100	250	151200	147.42
Integrated-with PAs	36600	189	100	300	94000	256.92

Table S5. Overall landscape connectivity of priority patches for different conservation objectives with three selected dispersal distances.

Conservation objectives	Dispersal distances		
	10 km	30 km	100 km
Biodiversity-with PAs	0.80	1.33	3.48
Landscape connectivity-with PAs	1.52	2.64	4.73
Ecosystem services-with PAs	1.83	2.60	4.38
Integrated-with PAs	1.05	1.82	3.81
